# The moderating role of an oxytocin receptor gene polymorphism in the relation between unsupportive social interactions and coping profiles: implications for depression

**DOI:** 10.3389/fpsyg.2015.01133

**Published:** 2015-08-11

**Authors:** Opal A. McInnis, Robyn J. McQuaid, Kimberly Matheson, Hymie Anisman

**Affiliations:** ^1^Department of Neuroscience, Carleton UniversityOttawa, ON, Canada; ^2^Department of Health Sciences, Carleton UniversityOttawa, ON, Canada

**Keywords:** depression, polymorphism, oxytocin, social support, coping, social interaction

## Abstract

Oxytocin is a hormone that is thought to influence prosocial behaviors and may be important in modulating responses to both positive and negative social interactions. Indeed, a single nucleotide polymorphism, rs53576, of the oxytocin receptor gene (OXTR) has been associated with decreased trust, empathy, optimism, and social support seeking, which are important components of coping with stressors. In the current study, conducted among undergraduate students (*N* = 225), it was shown that parental and peer social support was related to fewer depressive symptoms through elevated problem-focused coping and lower emotion-focused coping, and these effects were independent of the OXTR polymorphism. Unsupportive social interactions from parents were associated with more severe depressive symptoms through the greater use of emotion-focused coping, and this relation was moderated by the OXTR genotype. Specifically, individuals who carried the polymorphism on one or both of their alleles demonstrated increased emotion-focused coping following unsupportive responses compared to those without the polymorphism. Likewise, lower problem-focused coping mediated the relation between parental and peer unsupportive responses to depressive symptoms, but this mediated relation was only evident among carriers of the polymorphism. These findings suggest that carrying this OXTR polymorphism might favor disadvantageous coping styles in the face of negative social interactions, which in turn are linked to poor mood. Regardless of genotype, parental, and peer social support are fundamental in determining stress-related coping and well-being.

## Introduction

Supportive relationships and social connectedness are important predictors of health and well-being that serve as a buffer against several negative consequences of stressors (Cohen and Wills, 1985; Thoits, 2011). In contrast, a lack of social support has been associated with increased risk of chronic health conditions, such as heart disease and diabetes (House et al., 1988; Holt-Lunstad et al., 2010). Thus, enhancing social connectedness and social identity may attenuate depressive symptomatology (Cruwys et al., 2014, 2015). The experience of unsupportive social relationships, comprise negative, or ineffective social interactions, when help or advice is sought during a challenging or stressful time (Ingram et al., 1999, 2001). These unsupportive responses from others include the minimization of problems, blaming the individual, distancing themselves from an individual and their problems, and bumbling attempts to provide support. Importantly, the experience of unsupportive social interactions predicts depressive symptoms above and beyond the contribution of social support (Ingram et al., 1999; Song and Ingram, 2002). Despite the established beneficial effects of social support and the profound impact of unsupportive social interactions on well-being, the biological mechanisms underlying their influence remain largely unknown and under-investigated.

Oxytocin is a hormone that may contribute to a constellation of social behaviors, ranging from trust (Kosfeld et al., 2005) and attachment (Buchheim et al., 2009) to positive communication (Ditzen et al., 2009) and intergroup cooperation (De Dreu et al., 2010). The involvement of oxytocin in these prosocial behaviors in humans has been demonstrated following its administration through a nasal spray (Bakermans-Kranenburg and van IJzendoorn, 2013). As well, support for the involvement of oxytocin in mediating social behavior has come from genetic studies. Specifically, variations in the gene coding for the oxytocin receptor OXTR, in which a single nucleotide polymorphism (SNP) rs53576, which involves a guanine (G) to adenine (A) substitution, has been associated with diminished prosocial behaviors (Kumsta and Heinrichs, 2013). In this regard, compared to individuals who were homozygous for the G allele (i.e., the SNP was not present), A carriers tended to be less empathetic (Rodrigues et al., 2009), displayed lower parental sensitivity (Bakermans-Kranenburg and van IJzendoorn, 2008), and lower trust-related behaviors (Krueger et al., 2012). This SNP has also been associated with lower positive affect (Lucht et al., 2009), and self-esteem as well as greater depressive symptoms (Saphire-Bernstein et al., 2011). In effect, individuals who carry this SNP on one or both alleles (AG or AA genotype) appear to be less socially inclined and potentially at a greater risk for mental health disturbances.

Although coping strategies are not intrinsically negative or positive, depression is frequently associated with the endorsement of lower levels of problem-focused coping and higher levels of emotion-focused coping (Matheson and Anisman, 2003). For instance, depressive disorders have been tied to greater levels of rumination (Aldao et al., 2010) and emotional containment (Ravindran et al., 2002), as well as decreased social support seeking (Matheson and Anisman, 2003) and reduced use of cognitive restructuring (Ravindran et al., 2002). Given that A carriers are less apt to use social support as a means of coping, and benefit less from this coping method, it is possible that the presence of the OXTR SNP might favor the adoption of a relatively narrow range of effective coping strategies (i.e., those that do not rely on social support resources). As a result, the A allele might be associated with greater vulnerabilty to the negative impacts of stressors relative to those with the G allele.

There have been several reports, however, that do not comfortably align with the perspective that the A allele of the OXTR rs53576 gene is associated with vulnerability to disturbed social and emotional functioning. Indeed, the G allele of the OXTR was associated with greater social sensitivity (Bradley et al., 2011; McQuaid et al., 2013; Hostinar et al., 2014), which in the context of negative early life experiences, may be accompanied by greater emotional dysregulation (Bradley et al., 2011) and elevated depressive symptoms among adults (McQuaid et al., 2013). As well, maltreated adolescents who were homozygous for the G allele were more likely to perceive lower social support and reported greater internalizing of symptoms compared to maltreated A allele carriers (Hostinar et al., 2014). The social sensitivity perspective is in line with the suggestion that certain genetic variants may promote behavioral and emotional plasticity, so that environmental and experiential factors, irrespective of whether they are positive or negative, have greater effects on later outcomes (Belsky and Pluess, 2009; Belsky et al., 2009). In essence, the presence of the GG alleles might be accompanied by elevated sensitivity to social cues, irrespective of whether these involved a positive and nurturing early life environment or one that was more negative, and as a result influence social inclinations and mood in adulthood (Bradley et al., 2011; McQuaid et al., 2013; Hostinar et al., 2014).

The elevated sensitivity to environmental factors and the heightened neuroplasticity associated with increased oxytocin functioning (Lin et al., 2012) and with the G allele, could promote the adoption or development of social coping methods (McQuaid et al., 2014a). Indeed, within a stable or warm family environment, G carriers reported greater positive affect and ‘resilient’ coping, an association that was not observed among those with the AA genotype (Bradley et al., 2013). Conversely, those with the AA genotype sought less emotional social support during distress compared to G carriers (Kim et al., 2010), and also appeared to be less able to benefit from social support (Chen et al., 2011). Among adolescents who carried an A allele, but not among GG homozygotes, experiences of maternal depression predicted lower social functioning, which, in turn, was associated with elevated depressive symptoms (Thompson et al., 2014).

Although unsupportive relationships can have profound effects on mood states, it is uncertain whether the effects of such relationships vary as a function of oxytocin levels or the presence of the OXTR polymorphism. As well, coping methods (e.g., emotion-, avoidant-, and problem-focused coping) which are also important predictors of well-being have not been investigated in association with the genetic variants of the OXTR. In the present investigation we assessed experiences of social support and unsupport from both parents and peers in relation to depressive symptoms and whether these relations were mediated by coping styles. It was of particular interest to determine whether the OXTR rs53576 genotype moderated these mediated relationships. It is possible that the greater social sensitivity of those with the GG genotype would be accompanied by emotion-focused coping in response to unsupportive social interactions, and more effective coping skills in the presence of social support. In contrast, A carriers, who tend to have a more negative affect (and may be less sensitive to social interactions), might be more likely to adopt disadavantageous coping methods that involve emotion- more than problem-focused coping styles, irrespective of perceiving support, or experiencing unsupportive interactions.

## Materials and Methods

### Participants

Participants included 232 White/Euro-Caucasian female (*n* = 189) and male (*n* = 43) undergraduate students. Participants were recruited through a university online-recruitment system as well as through campus advertisements. Ages ranged between 17 and 35 years of age (*M* = 19.75, SD = 2.78). Current living arrangements varied, with the majority of participants living with either friends/roommates (52.16%), or with parents (31.47%), and the remaining participants reporting living alone (5.60%), with a significant other (4.74%), or other arrangements (6.03%; e.g., living with children).

### Procedure

Following the provision of informed consent, participants were provided with a series of questionnaires that assessed demographic information, current symptoms of depression, coping styles, as well as levels of perceived support and unsupportive interactions from parents and peers. Following completion of questionnaires, a single saliva sample was collected from participants for DNA analyses. All participants were provided with a written debriefing explaining the purpose and objectives of the study, as well as researcher contact information. All procedures for the present study were approved by the Carleton University Ethics Committee for Psychological Research.

### Genotyping

Saliva samples for DNA analyses were collected using an Oragene OG-500 saliva sample collection kit purchased from DNA Genotek (Ottawa, ON, Canada). Manufacturer’s instructions were followed for the extraction of genomic DNA and following extraction samples were diluted to approximately equal concentrations (20 ng/μL). DNA samples were genotyped using quantitative polymerase chain reaction (qPCR). The amplification reactions were performed using approximately 1 μL (20 ng) of genomic template, 0.6 μL of each primer (with a concentration of 10 μM), 1.2 μL of dNTP, 1.5 μL of 10X buffer, 1.5 μL of MgCl_2_, 0.3 μL of Salmon Sperm DNA, 0.15 μL of Taq polymerase, 0.015 of SYBR green, 8.135 μL of water. The total volume of the resulting solution was 15 μL. Solutions were plated in duplicate and qPCR products were run on 2% agarose gel electrophoresis to visualize and confirm qPCR results. The primer sequences used for qPCR were the following:

OXTR F1 forward: TCCCTGTTTCTGTGGGACTGAGGAC
OXTR F2 forward: TCCCTGTTTCTGTGGGACTGAGGAT
OXTR reverse: TCCCTGTTTCTGTGGGACTGAGGAT

Allele distribution for the OXTR polymorphism comprised 104 individuals with the homozygote GG genotype, (87 female, 17 male), 89 individuals with the heterozygote AG genotype (71 female, 18 male), and 32 individuals with the homozygote AA genotype (25 female, 7 male). Genotype distributions did not differ as a function of gender χ^2^_(1)_ = 0.73, *p* = 0.70. Additionally, genotype distributions for males, χ^2^_(1)_ = 0.35, *p* = 0.55, and females, χ^2^_(1)_ = 2.79, *p* = 0.09, met Hardy–Weinberg Equilibrium expectations. The initial sample size was 232 but there were seven individuals for whom the genotype could not be determined and hence they were excluded from any subsequent analyses making the overall *N* = 225. Further, due to the infrequency of the AA genotype, a dominant model was used wherein all A carriers (AA and AG were pooled) were compared to individuals with the GG genotype.

### Measures

#### Depressive Symptoms

Depressive symptoms were assessed using the Beck Depression Inventory (BDI; Beck et al., 1961). This is a 21-item questionnaire in which participants respond to each item by selecting one of four options that range from low to high depression symptomology. The scores were calculated as the total sum across all items (Cronbach’s α = 0.90).

#### Unsupportive Social Interactions

Levels of unsupportive social interactions from parents and peers were assessed using the Unsupportive Social Interactions Inventory (USII; Ingram et al., 2001). This 24-item scale was administered twice (once for parents, and once for peers) and assessed the degree of perceived unsupport individuals received from their parents or peers when turning to them during a recent stressful or challenging time. Participants responded to each item ranging from none (0) to a lot (4). The unsupport scale comprised four subscales that included distancing (behavioral or emotional disengagement; e.g., “Would not seem to want to hear about it”), bumbling (behaviors that are awkward, or uncomfortable; e.g., “Would try to cheer me up when I was not ready to”), minimizing (attempts to minimize the individual’s concerns; e.g., “Would feel that I was overreacting”), and blaming (finding fault or criticism; e.g., “Would make “I told you so” or similar comments”). The four subscales were highly correlated with one another [ranging from *r* = 0.47 to 0.65 (Parents) and *r* = 0.42 to 0.58 (Peers)], and so total mean scores of unsupport were used (Peers: Cronbach’s α = 0.92; Parents: Cronbach’s α = 0.93).

#### Social Support

Perceived social support from parents and peers was assessed using the Social Provisions Scale (Cutrona and Russell, 1987). Participants were asked to respond to this shortened 12-item scale twice (once for parents, and once for peers) by rating the degree to which their parents or peers are currently providing them with different forms of support including, guidance, reassurance of worth, reliable alliance, social integration, opportunity to provide nurturance and attachment. This shortened version has been shown to demonstrate good construct validity (Russell et al., 1984). Total mean scores of social support were used and demonstrated good reliability (Peers: Cronbach’s α = 0.87; Parents: Cronbach’s α = 0.81).

#### Coping Styles

The Survey of Coping Profile Endorsement (Matheson and Anisman, 2003) is a 50-item scale that assesses the means individuals use to cope. Participants indicated on a scale of never (1) to almost always (5), the extent to which they would use the behavior as a way of dealing with problems or stressors in recent weeks. A principal component analysis (PCA) with a varimax rotation was conducted to determine the underlying factor structure of this scale. The PCA was performed on 13 subscales based on earlier studies (Matheson and Anisman, 2003) and were included on a factor when loadings were greater than 0.40. Three factors emerged which encompassed emotion-, avoidant-, and problem-focused coping. The factor loadings were similar to that of previous findings (Raspopow et al., 2013; McQuaid et al., 2014b) and Cronbach’s alphas for the three factors confirmed that they were well-constructed. Emotion-focused coping comprised ruminations, emotional expression, blaming others, self-blame, and wishful thinking (Cronbach’s α = 0.90). Avoidant coping comprised, cognitive distraction, passive resignation, and emotional containment (Cronbach’s α = 0.82). Problem-focused coping comprised problem solving, cognitive restructuring, active distraction, humor, and social support seeking (Cronbach’s α = 0.85).

### Statistical Analyses

The statistical analyses were performed using IBM SPSS Statistics 20 for Windows (Armonk, NY, USA: IBM Corp.). Independent samples *t*-tests were performed to assess differences of OXTR and gender on scores of depression, coping, and experiences of unsupportive social interactions as well as, social support. Pearson correlation scores were calculated to assess the relations between self-reported scores for depression, unsupportive social interactions, social support, and coping. Moderated mediation analyses were conducted using bootstrapping procedures and confidence intervals based on 5000 resamples (Preacher et al., 2007). Unstandardized scores were used for all regression analyses. In the moderated mediation analyses OXTR genotype was treated as the moderator, unsupport or social support were used as independent variables, coping styles as mediator variables and depressive symptoms as the outcome.

## Results

There were no differences as a function of individuals’ genotype on depression [*t*(1,223) = -0.04, *p* = 0.97], perceived social support from parents [*t*(1,223) = 1.14, *p* = 0.26] or peers [*t*(1,223) = -0.38, *p* = 0.70], or unsupport from parents [*t*(1,223) = -0.06, *p* = 0.95] or peers [*t*(1,223) = -0.54, *p* = 0.59]. Likewise, differences were not observed across genotypes with respect to emotion-focused [*t*(1,223) = 0.37, *p* = 0.71], avoidant-focused [*t*(1,223) = 0.77, *p* = 0.44], or problem-focused coping [*t*(1,223) = -0.38, *p* = 0.70; see **Table 1** for descriptives]. Analyses were also conducted to determine if any of the variables of interest varied as a function of gender. In this regard, reported depressive symptoms were higher among females, *t*(1,91) = 4.56, *p* < 0.001, as were reports of emotion- and avoidant-focused coping, *t*(1,85) = 4.24, *p* < 0.001, and *t*(1,230) = 2.23, *p* < 0.05, respectively (see **Table 2** for all descriptives and *t*-test values).

**Table 1:** Mean, SD, and range for study variables by oxytocin receptor gene (OXTR) rs53576 genotype.

	GG	AG	AA	Overall
Beck depression inventory	*M* = 9.18 ± 7.55 Range: 0–31.00	*M* = 9.08 ± 8.31 Range: 0–35.50	*M* = 9.61 ± 9.18 Range: 0–33.00	*M* = 9.15 ± 8.01 Range: 0–35.50
Social support (Parents)	*M* = 3.22 ± 0.49 Range: 1.29–4.00	*M* = 3.30 ± 0.59 Range: 1.29–4.00	*M* = 3.11 ± 0.75 Range: 1.42–4.00	*M* = 3.24 ± 0.57 Range: 1.29–4.00
Social support (Peers)	*M* = 3.46 ± 0.35 Range: 2.00–4.00	*M* = 3.43 ± 0.47 Range: 1.85–3.92	*M* = 3.30 ± 0.58 Range: 1.87–4.00	*M* = 3.42 ± 0.44 Range: 1.85–4.00
Unsupport (Parents)	*M* = 1.40 ± 0.76 Range: 0–3.50	*M* = 1.40 ± 0.82 Range: 0.08–3.50	*M* = 1.43 ± 0.94 Range: 0.38–3.50	*M* = 1.40 ± 0.80 Range: 0–3.50
Unsupport (Peers)	*M* = 1.20 ± 0.59 Range: 0.17–3.25	*M* = 1.24 ± 0.63 Range: 0.08–2.88	*M* = 1.27 ± 0.74 Range: 0.17–3.13	*M* = 1.23 ± 0.63 Range: 0.08–3.25
Emotion-focused coping	*M* = 1.92 ± 0.80 Range = 0–3.80	*M* = 1.86 ± 0.76 Range: 0.15–3.47	*M* = 1.94 ± 0.80 Range: 0.57–3.25	*M* = 1.90 ± 0.77 Range: 0–3.80
Avoidance-focused coping	*M* = 2.09 ± 0.65 Range: 0.64–3.50	*M* = 2.01 ± 0.67 Range: 0.33–3.39	*M* = 2.07 ± 0.64 Range: 0.67–3.28	*M* = 2.06 ± 0.65 Range: 0.33–3.50
Problem-focused coping	*M* = 2.55 ± 0.53 Range: 1.02–3.69	*M* = 2.56 ± 0.58 Range: 0.80–3.72	*M* = 2.51 ± 0.65 Range: 1.34–3.61	*M* = 2.56 ± 0.65 Range: 0.80–3.90

**Table 2:** Mean, SD, and *t*-test values of study variables by gender.

	Males	Females	*t*-test values
Beck depression inventory	*M* = 5.33 ± 5.47	*M* = 10.02 ± 8.23	*t*(1,92) = 4.56, *p* < 0.001
Social support (Parents)	*M* = 3.37 ± 0.43	*M* = 3.21 ± 0.60	*t*(1,89) = -2.02, *p* < 0.05
Social support (Peers)	*M* = 3.45 ± 0.42	*M* = 3.42 ± 0.44	*t*(1,230) = -0.47, *p* = 0.64
Unsupport (Parents)	*M* = 1.07 ± 0.54	*M* = 1.47 ± 0.84	*t*(1,94) = 3.98, *p* < 0.001
Unsupport (Peers)	*M* = 1.23 ± 0.47	*M* = 1.22 ± 0.66	*t*(1,84) = -0.17, *p* = 0.86
Emotion-focused coping	*M* = 1.54 ± 0.56	*M* = 1.98 ± 0.79	*t*(1,85) = 4.24, *p* < 0.001
Avoidance-focused coping	*M* = 1.86 ± 0.62	*M* = 2.10 ± 0.65	*t*(1,230) = 2.23, *p* < 0.05
Problem-focused coping	*M* = 2.59 ± 0.50	*M* = 2.56 ± 0.59	*t*(1,230) = -0.28, *p* = 0.78

*When Levene’s Test of Equality of Variances was violated equal variances not assumed are reported.*

As expected, depression scores were positively correlated with unsupportive relations from parents (*r* = 0.59, *p* < 0.001) and peers (*r* = 0.44, *p* < 0.001), and negatively related to social support from parents (*r* = -0.62, *p* < 0.001) and peers (*r* = -0.47, *p* < 0.001). As predicted as well, depressive symptoms were positively related to emotion-focused coping (*r* = 0.62, *p* < 0.001) and avoidant-focused coping (*r* = 0.41, *p* < 0.001), whereas problem-focused coping was negatively associated with depression scores (*r* = -0.43, *p* < 0.001; **Table 3**).

**Table 3:** Relations between depressive symptoms, social support, unsupport, and coping.

	1	2	3	4	5	6	7
(1) Social support (Parents)							
(2) Social support (Peers)	0.39^∗∗∗^						
(3) Unsupport (Parents)	-0.65^∗∗∗^	-0.31^∗∗∗^					
(4) Unsupport (Peers)	-0.30^∗∗∗^	-0.48^∗∗∗^	0.61^∗∗∗^				
(5) Emotion-focused coping	-0.37^∗∗∗^	-0.34^∗∗∗^	0.46^∗∗∗^	0.45^∗∗∗^			
(6) Avoidant-focused coping	-0.25^∗∗∗^	-0.25^∗∗∗^	0.35^∗∗∗^	0.32^∗∗∗^	0.51^∗∗∗^		
(7) Problem-focused coping	0.40^∗∗∗^	0.51^∗∗∗^	-0.26^∗∗∗^	-0.21^∗∗^	-0.16^∗^	-0.10	
(8) Depressive symptoms	-0.62^∗∗∗^	-0.47^∗∗∗^	0.59^∗∗∗^	0.44^∗∗∗^	0.62^∗∗∗^	0.41^∗∗∗^	-0.43^∗∗∗^

*^∗^p < 0.05, ^∗∗^p < 0.01, ^∗∗∗^p < 0.001.*

### Parental Support and Unsupport

It was of interest to examine the influence of OXTR genotype on the mediated relations between parental social support, unsupport, and depressive symptoms through coping styles. Preliminary analyses revealed that avoidant-focused coping was not an important mediator of these relations [95% CI (-0.12,0.64)], and was thus excluded from subsequent analyses examining the moderating role of OXTR genotype. Moderated multiple mediation analyses were performed using bootstrapping techniques and confidence intervals based on 5000 iterations (Preacher et al., 2007), in which we assessed whether the association between parental social support and depressive symptoms mediated by problem- as well as emotion-focused coping was moderated by the OXTR genotype. In particular, it was tested whether the OXTR genotype moderated the path between social support and coping styles.

These analyses revealed that the OXTR genotype did not moderate the mediating role of problem-focused coping (*b* = 0.03, *t* = 0.20, *p* = 0.83) or emotion-focused coping (*b* = -0.12, *t* = -0.68, *p* = 0.50) on the relations between levels of social support from parents and depressive symptoms. In effect, regardless of the genotype, social support was related to depressive affect and this was mediated by greater problem- and lower emotion-focused coping [95% CI (-1.91,-0.58), 95% CI (-2.98,-1.39), respectively]. Alternative models assessing whether OXTR moderated the association between both problem- and emotion-focused coping on depressive symptoms were found not to be significant.

**FIGURE 1:**
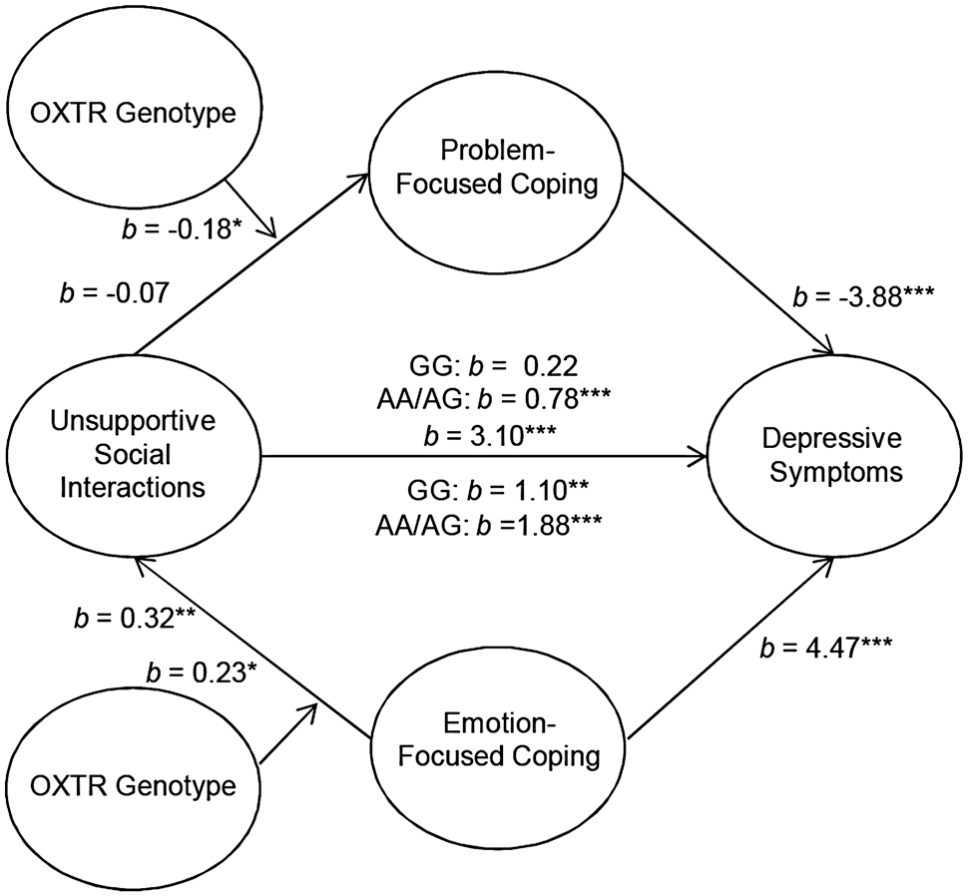
**Schematic of the moderated multiple mediation analyses examining parental unsupport.** The relation between unsupportive social interactions from parents and depressive symptoms through problem-focused coping was moderated by oxytocin receptor gene (OXTR) rs53576 genotype, such that it was only significant among A carriers. As well, the relation between unsupportive responses from parents and depressive symptoms through emotion-focused coping as moderated by OXTR genotype. This mediated model was significant irrespective of genotype, but was stronger among A carriers. ^∗^*p* ≤ 0.05, ^∗∗^*p* < 0.01, ^∗∗∗^*p* < 0.001.

Although OXTR genotype did not influence the mediating role of coping between parental social support and depressive symptoms, it was of interest to examine the moderating role of OXTR genotype in the context of unsupportive social interactions. Analyses were performed to determine the moderating influence of OXTR on the association between unsupport from parents and problem-focused coping to predict depressive symptoms. These analyses revealed that the OXTR genotype moderated the mediating role of problem-focused coping on the relation between levels of unsupport from parents and depressive symptoms *b* = -0.18, *t* = -1.96, *p* = 0.05. Specifically, unsupportive interactions with parents were associated with higher depressive symptoms and this was mediated through lower problem-focused coping. However, this mediated relationship was only present among individuals who carried an A allele [95% CI (0.42,1.70)] and, as expected, was absent among those with the GG genotype [95% CI (-0.25,0.80)] for the OXTR gene (**Figure 1**). Moreover, the OXTR genotype moderated the mediating role of emotion-focused coping in the relation between unsupport from parents and depressive symptoms, *b* = 0.23, *t* = 1.94, *p* = 0.05. Perceptions of unsupportive relations were associated with higher emotion-focused coping, which, in turn was related to higher depressive symptoms. Unlike problem-focused coping, this mediated relationship was observed irrespective of the OXTR genotype, but was stronger among A allele carriers [95% CI (1.73,3.22)] compared to individuals with the GG genotype [95% CI (0.54,2.35)] (**Figure 1**). It should be noted that the moderated effect of the OXTR polymorphism was small, and thus at this juncture the results should be interpreted cautiously. Once again, alternative models assessing whether OXTR moderated the path between both problem- and emotion-focused coping on depressive symptoms were not significant.

### Peer Support and Unsupport

**FIGURE 2:**
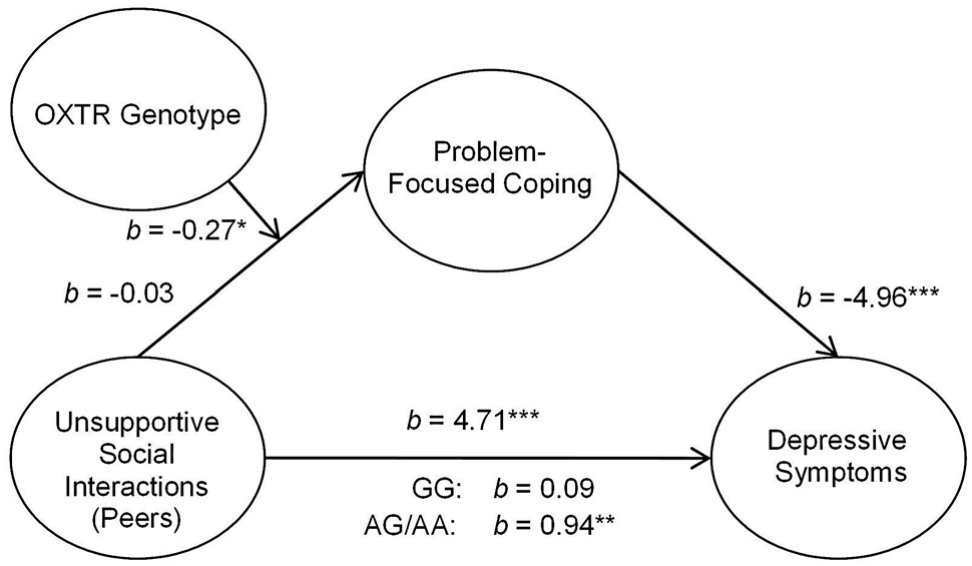
**Schematic of the moderated mediation analyses examining peer unsupport.** The relation between unsupportive social interactions from peers and depressive symptoms through problem-focused coping was moderated by OXTR rs53576 genotype, only being significant among A carriers. ^∗^*p* < 0.05, ^∗∗^*p* < 0.01, and ^∗∗∗^*p <* 0.001.

In addition to assessing the associations between OXTR and unsupportive responses from parents, we examined the relation between unsupport from peers and coping styles as well as between social support from peers and coping styles. As observed with social support from parents, peer support in relation to depressive symptoms through coping styles was not moderated by the OXTR genotype. Indeed, peer support was important regardless of genotype such that greater levels of perceived peer social support were associated with greater problem- and lower emotion-focused coping and this was related to lower depressive symptoms [problem-focused: 95% CI (-3.93,-1.29); emotion-focused: 95% CI (-4.82,-2.02)]. Furthermore, the OXTR genotype did not moderate the mediated relation between unsupport from peers and depressive symptoms through emotion-focused coping, *b* = 0.07, *t* = 0.49, *p* = 0.63. In contrast, the OXTR genotype moderated this relation when problem-focused coping was considered as a mediator, *b* = -0.27, *t* = -2.20, *p* < 0.05. This mediated relation was observed among A allele carriers (**Figure 2**), but was entirely absent among those with the GG genotype^1^.

Due to the potential influence of gender on some of the factors assessed in the moderated mediation analyses (i.e., emotion-focused coping and depressive symptoms) the data were re-analyzed using gender as a covariate. These analyses revealed similar results, in that OXTR remained a non-significant moderator of models in which social support was used as the independent variable. As well, the moderated effect of the OXTR genotype on the relation between peer unsupport and problem-focused coping and depressive symptoms remained unchanged. When examining the moderated effect of OXTR in models where parental unsupport was used as the independent variable, the *p*-value for problem-focused coping was reduced. However, the moderated effect of OXTR on emotion-focused coping changed marginally from *p* = 0.05 to *p* = 0.07. The overall direction of relationships remained unchanged.

## Discussion

The current findings revealed that the OXTR polymorphism rs53576 moderated the association between unsupportive social interactions from parents and peers and problem-focused coping responses in their relation to depressive scores. Specifically, this mediated relation was evident in A carriers, but absent among those with the GG genotype. It seems that in the presence of the A allele it was less likely that individuals would adopt problem-focused strategies in the face of unsupportive interactions, which could potentially contribute to depressive disorders. The current findings also indicated that the adoption of emotion-focused coping in association with perceived unsupportive parental responses was tied to greater depressive symptoms, and this was particularly notable among A carriers. It is uncertain why this heightened relation existed. It is possible that diminished reliance on social support seeking among A carriers was accompanied by exaggerated emotion-focused coping efforts under conditions of unsupportive responses. In line with these findings, adolescents who carried the A allele for the OXTR rs53576 reported greater levels of loneliness if they also perceived their social network more negatively (van Roekel et al., 2013). The present findings are consistent with those indicating that depressive mood is accompanied by elevated emotion-focused coping at the expense of problem-focused coping (Matheson and Anisman, 2003). Whether this reflects actions of coping on depression, altered coping secondary to depression, or variations in the sensitivity to social cues, it is uncertain given the correlational nature of the present data.

It is somewhat puzzling that the relation between peer unsupport and emotion-focused coping was present irrespective of genotype, whereas this relationship was moderated by the OXTR genotype in the context of parental unsupport. However, for individuals in this age group, responses from peers may be especially significant (Wilkinson, 2004) and hence regardless of genotype, peer unsupport may be highly linked to emotion-focused coping. This speaks to the fact that the effects of social interactions on coping and well-being are not all similarly influenced by genetic predispositions.

The current findings indicated that perceptions of both parental and peer *social support* were associated with depressive symptoms through emotion- and problem-focused coping. Moreover, these relations were not influenced by the oxytocin genotype, which contrasts with the pattern observed with respect to unsupportive social interactions. Social support is fundamental to well-being and it is possible that in relation to coping styles, differences related to genotype are less marked. This said, there have been reports of social support interacting with the OXTR genotype, indicating that in comparison to individuals with the AA genotype, G carriers of the OXTR rs53576 exhibited diminished stress responses (i.e., decreased cortisol) when social support was available (Chen et al., 2011). In the present investigation, however, the interaction with the OXTR polymorphism was limited to unsupportive relations and was not apparent with respect to social support. Follow-up statistical analyses indicated that the lack of an association of the OXTR polymorphism with social support and coping was apparent irrespective of whether or not AG carriers were pooled with the AA or GG genotypes. However, the small number of AA individuals in the analyses makes it necessary for further replication to determine the relation (or lack of it) between the OXTR polymorphism, social support and coping styles.

Finally, the current data are consistent with previous studies that linked both unsupport and coping styles with depressive symptoms (Ingram et al., 1999; Raspopow et al., 2013; McQuaid et al., 2014b), and these relations were more apparent among A carriers. Although these data are in line with the view that the A allele is a vulnerability factor in relation to depressive symptoms, they are not consistent with the social sensitivity hypothesis that G allele carriers are more sensitive, rendering them more susceptible to the consequences of a negative environment (Bradley et al., 2011; McQuaid et al., 2013; Hostinar et al., 2014). It is possible, however, that the relationship between particular genotypes and negative events might vary developmentally. In particular, the heightened social sensitivity associated with the G allele of the OXTR rs53576 was more closely aligned with mood symptoms when the negative social interactions were experienced early in life, as in the case of childhood abuse or neglect (Bradley et al., 2011; McQuaid et al., 2013; Hostinar et al., 2014). It should be added that the nature of unsupportive social interactions experienced among adults differs appreciably from that of childhood maltreatment, and thus a comparison of these stressful experiences may be inappropriate. Furthermore, it is possible that the link between oxytocin functioning and social sensitivity may vary with specific contextual conditions. For instance, oxytocin might have prosocial effects in a test involving positive social behaviors, but might have very different actions in situations involving social exclusion or ostracism. We observed that G carriers were more sensitive to the effects of an acute experience of social ostracism, although it is uncertain whether these same individuals would be more likely to adopt social support seeking as a primary coping strategy (McQuaid et al., 2015).

Although the present study indicated an association of the A allele with seemingly less productive coping processes, there are several limitations that should be considered. The modest sample size and the number of variables examined may be problematic in a gene-association study (Ohashi and Tokunaga, 2001), and thus the present findings ought to be considered as being provisional, pending a replication of this study. Also, due to the limited number of participants, we were unable to examine the relative risk for negative mood outcomes across the three OXTR genotypes. Examination of the genotypes separately can be particularly informative and the choice to collapse and use a dominant model may not always be appropriate. For example, following a social stressor that comprised social ostracism, when assessing psychosocial measures we observed that responses of participants with the heterozygote AG genotype for the OXTR rs53576 aligned more closely to those with the AA genotype, whereas on physiological measures (cortisol and blood pressure) the heterozygotes displayed profiles that were more similar to individuals with the GG genotype (McQuaid et al., 2015). In the present investigation, the choice to combine individuals carrying the AA and AG alleles was predicated on earlier studies examining this OXTR SNP (Bakermans-Kranenburg and van IJzendoorn, 2008; Rodrigues et al., 2009; Saphire-Bernstein et al., 2011; Krueger et al., 2012), although a meta-analysis failed to detect a significant combined effect of the OXTR rs53576 polymorphism on social behaviors (Bakermans-Kranenburg and van Ijzendoorn, 2014). However, this does not imply that alternative analytic approaches are inappropriate. Ultimately, evaluating the three genotypes independently, despite the low incidence of the AA genotype (∼15% in Euro-Caucasians), would be ideal.

Males and females differed on several dimensions (e.g., depressive symptoms, emotion-and avoidance-focused coping, parental unsupport and support), but these differences did not vary as a function of the OXTR genotype. As the sample largely comprised females (∼80%) and only a modest number of males were assessed, the contribution of the OXTR genotype to these gender differences warrants further research. This is especially the case as oxytocin may interact with estrogen and with menstrual cycle (Choleris et al., 2003), and it is possible that relations between behavior and the OXTR genotype might also vary with menstrual cycle. However, when gender was treated as a covariate the moderated effect of the OXTR genotype became less significant when examining parental unsupport to depressive symptoms through emotion-focused coping. Further, due to the cross-sectional nature of the study the directionality of the variables of interest is not known. This greatly limits the interpretation of the mediation analyses, and as such, inferences about temporal relations between the variables cannot be inferred. The possibility remains that participants’ current depressive symptoms could have biased their perceptions of unsupportive social interactions and social support. Finally, although there have been several studies linking the OXTR rs53576 gene polymorphism to prosocial behaviors, the functionality of this polymorphism is uncertain (i.e., whether this SNP actually disturbs the receptors responsivity; Inoue et al., 1994). Nevertheless, it has been suggested that this polymorphism may contribute to the suppression of the protein making up these receptors (i.e., transcription suppression) and hence the presence of these receptors themselves (Mizumoto et al., 1997).

Despite the limitations, the present findings are consistent with the view that A carriers may be more susceptible to negative mood outcomes through the use of less effective coping methods. Yet, the link to psychological disorders, such as depression, is exceedingly complex, especially as genetic factors that are beneficial in certain environments, particularly those that involve social interactions, may be unfavorable in others.

## Author Contributions

OM, RM, and HA contributed to the inception and design of the current experiment. Testing and data collection were performed by OM and RM. The processing of samples was performed by OM and RM. Data analysis and the writing of the manuscript were performed by OM, RM, KM, and HA. All authors approved the final version of the paper for submission.

## Conflict of Interest Statement

The authors declare that the research was conducted in the absence of any commercial or financial relationships that could be construed as a potential conflict of interest.

## Footnotes

1 As the BDI scores were positively skewed (skewness *z* = 6.48), additional analyses were undertaken of the square root transformed BDI scores. The results of this analysis fully mapped on to that using the non-transformed data.
